# Regulation of plant antiviral defense genes via host RNA-silencing mechanisms

**DOI:** 10.1186/s12985-021-01664-3

**Published:** 2021-09-26

**Authors:** Paola Leonetti, Johannes Stuttmann, Vitantonio Pantaleo

**Affiliations:** 1 Department of Biology, Agricultural and Food Sciences, Institute for Sustainable Plant Protection, Research Unit of Bari, CNR, 70126 Bari, Italy; 2 Institute of Biology, Department of Plant Genetics, Martin Luther University, Halle-Wittenberg, 06120 Halle (Saale), Germany; 3 Institute of Biochemistry and Biotechnology, Martin Luther University, Halle-Wittenberg, 06120 Halle (Saale), Germany

**Keywords:** Resistance, dsRNAs, VAMPs, PRRs, NLRs, Broad-spectrum resistance, RNA silencing

## Abstract

**Background:**

Plants in nature or crops in the field interact with a multitude of beneficial or parasitic organisms, including bacteria, fungi and viruses. Viruses are highly specialized to infect a limited range of host plants, leading in extreme cases to the full invasion of the host and a diseased phenotype. Resistance to viruses can be mediated by various passive or active mechanisms, including the RNA-silencing machinery and the innate immune system.

**Main text:**

RNA-silencing mechanisms may inhibit viral replication, while viral components can elicit the innate immune system. Viruses that successfully enter the plant cell can elicit pattern-triggered immunity (PTI), albeit by yet unknown mechanisms. As a counter defense, viruses suppress PTI. Furthermore, viral Avirulence proteins (Avr) may be detected by intracellular immune receptors (Resistance proteins) to elicit effector-triggered immunity (ETI). ETI often culminates in a localized programmed cell death reaction, the hypersensitive response (HR), and is accompanied by a potent systemic defense response. In a dichotomous view, RNA silencing and innate immunity are seen as two separate mechanisms of resistance. Here, we review the intricate connections and similarities between these two regulatory systems, which are collectively required to ensure plant fitness and resilience.

**Conclusions:**

The detailed understanding of immune regulation at the transcriptional level provides novel opportunities for enhancing plant resistance to viruses by RNA-based technologies. However, extensive use of RNA technologies requires a thorough understanding of the molecular mechanisms of RNA gene regulation. We describe the main examples of host RNA-mediated regulation of virus resistance.

## Background

Plants encounter numerous microorganisms and other higher living organisms throughout their life span. Some are beneficial, and even symbiotic. Others are harmful and can cause disease and death of the host if a prompt defense reaction or immunity is not triggered. The resilience of plants to environmental conditions and changes depends on their ability to promote beneficial interactions and activate defenses, when necessary, in a robust and energy-efficient manner. Based on this, the numerous mechanisms of molecular signal perception which are similar, yet distinct in downstream responses, during interactions with symbionts and pathogens, therefore require a coordinated machinery of control [1].

Viruses are potential plant pathogens and, therefore, will encounter plant's defense barriers at every step of their replication cycle; i.e. via (i) spread in the agro-ecosystems and transmission, (ii) plant cell infection, and (iii) systemic invasion. In regards to the host, the mechanisms against viral attacks can be summarized in: (i) non-host resistance via physical barriers (e.g., waxy cuticles and/or thickened cell walls; preventing transmission by insect vectors), (ii*)* passive resistance in which the host blocks or lacks a component required by the virus to complete its life cycle, (iii) pattern-triggered immunity (PTI), (iv) effector-triggered immunity (ETI), and (v*)* the RNA-silencing system. Several RNA-based regulatory mechanisms of genes involved in resistance to microbes have been identified so far [2]. RNA-based mechanisms offer the advantage of being readily reversible in absence of pathogens. RNA-based regulation has emerged as a critical layer of control in plant immunity also in the case of virus infection. This review presents an overview of RNA-based regulatory mechanisms as the main actors of plant antiviral immune responses.

### PTI‐based antiviral responses

Canonical PTI is mediated by cell surface-localized pattern-recognition receptors (PRRs) that are either receptor-like kinases (RLKs) or receptor-like proteins. PRRs commonly recognize relatively conserved signature molecules characteristic for a whole class of organisms, referred to as microbe/pathogen‐associated molecular patterns (MAMPs/PAMPs; reviewed in [3]). The best-characterized PAMP-PRR pair involves recognition of a 22-amino-acid epitope (flg22) derived from bacterial flagellin by the leucine-rich repeat (LRR) receptor kinase FLAGELLIN-SENSING 2 (FLS2). PAMP recognition triggers a cascade of reactions collectively forming the basal defense layer. PTI-induced reactions include Ca^2+^-influx, activation of mitogen-activated protein kinases, production of reactive oxygen species (ROS) and nitric oxide (NO), cell wall reinforcement, and salicylic acid (SA) synthesis and signalling [4, 5]. Furthermore, PRRs may also detect host endogenous molecules released upon cell damage called damage-associated molecular patterns (DAMPs). During infection, many microbes translocate virulence factors (effectors) directly into the plant cell cytoplasm. One major function of effectors is the suppression of PTI responses, thus creating effector-triggered susceptibility [6]

Based on the firmly established definitions of microbial PAMPs and effectors [6, 7] viruses are not generally considered as encoding PAMPs or effectors. Nonetheless, successful transmission and entry into a plant cell exposes a virus to the PTI defense layer, and recent reports suggest that classical plant PTI initiated by transmembrane PRRs limits virus infection. Pre-activation of PTI with non‐viral PAMPs confers resistance to virus infection [8, 9], indicating that PTI‐induced immune responses confer protection against viruses, and most viral genomes encode suppressors of PTI, similar to microbial effectors, i.e. the disease-specific protein of rice stripe virus (RSV) [10], cauliflower mosaic virus (CaMV) P6 [11] and plum pox virus capsid protein [12]. Upon PAMP perception, PTI signalling depends on an intricate network of co-receptors and receptor-like cytoplasmic kinases (reviewed, in [13]), such as the LRR-RLK BRASSINOSTEROID INSENSITIVE1 (BRI1)‐ASSOCIATED KINASE 1 (BAK1/SERK3) and related SOMATIC EMBRYOGENESIS RECEPTOR KINASES (SERKs). As exclusively intracellular pathogens, how viruses might be detected extracellularly by the surface-localized PRRs, remains an open question. However, it is conceivable that, similar to DAMPs, viral PAMPs (VAMPs) are exposed in the apoplast either actively or upon cellular damage. In line with such a scenario, the detection of protein and RNA components of turnip mosaic virus (TuMV) in the plant extracellular space during viral infection was reported [14]; this would partly solve the apparent contradiction and open novel avenues of investigations in plant virology.

Similarly, classical PRRs mediating resistance to viruses and corresponding VAMP-ligands remain to be identified. However, double-stranded (ds) RNAs, dsRNA-like molecules of viral origin (i.e. replication intermediates, highly structured single stranded RNA transcripts), the synthetic dsRNA analog polyinosinic–polycytidylic acid and bacterial RNA preparations can induce PTI responses in *Arabidopsis* [15, 16]. Intriguingly, dsRNA-induced PTI is independent of *Dicer-like* proteins (DCLs), but requires the LRR-RLK SERK1 [15], thus making it distinct from the well-characterized PTI induced by microbial elicitors and the RNA-silencing pathway. Furthermore, NSP-INTERACTING KINASE 1 (NIK1), which belongs to the same class of LRR-RLKs as BAK1, is strongly associated to resistance to begomoviruses, and is targeted by the viral nuclear shuttle protein during infection [17]. Constitutive activation of NIK1 leads to repression of genes coding for components of the translation machinery and global suppression of translation. Thus, although NIK1 is structurally related BAK1/SERK3, it induces an immune response distinct from those depending on this major PRR co-receptor to suppress viral replication.

**Fig. 1:**
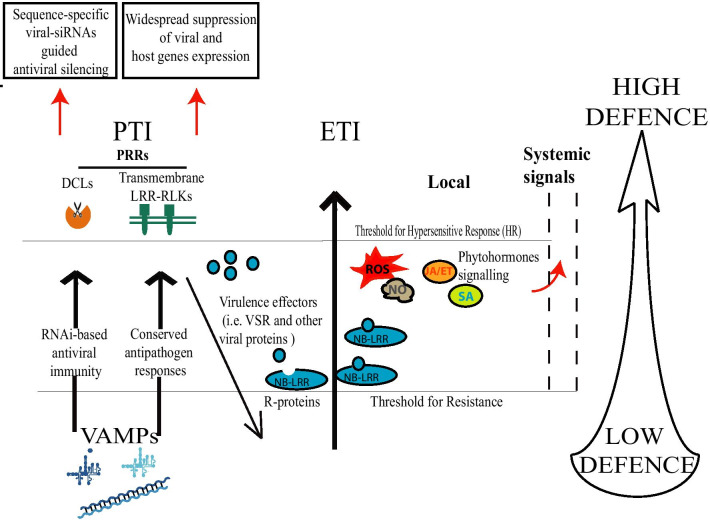
"Zig-Zag" model by Jones and Dangl [6] adapted for representing plant defense responses to viruses. Virus-associated molecular patterns, VAMPs, typically double-stranded (ds)RNA of viral origin, can induce either RNAi-based antiviral or a canonical pathogen triggered immunity (PTI) upon recognition by Dicer-like proteins (DCLs) or transmembrane leucine rich pattern-recognition receptors (PRRs), respectively. The PTI response evolves into sequence-specific antiviral silencing and/or into wide-spread suppression of host gene expression. Viruses express virulence effectors (i.e., viral suppressors of RNA silencing, VSRs) that can suppress PTI and lower the level of resistance. In resistant plant accessions, virulence effectors or virus structural components are recognised by intracellular immune receptors, commonly of the NB-LRR type. Immune receptor activation triggers production of reactive oxygen species (ROS), NO, phytohormone signalling (Jasmonate, JA; Ethylene, ET; salicylic acids, SA) and the hypersensitive responses (HR). ETI further induces systemic acquired resistance (SAR)

Although dsRNA can thus apparently induce PTI, it also triggers the classical RNA-silencing pathway, which acts as the major virus resistance mechanism in plants. Comparing PTI and the RNA-silencing pathway, DCLs function in analogy to PRRs by binding dsRNA molecules, the VAMPs, in the cytoplasm (Fig. 1) [18]. Activation of DCL-dependent defense is associated with a massive production of endogenous viral-activated siRNAs (va-siRNAs) and a consequent widespread silencing of host genes [19, 20]. Accordingly, classical PTI and the RNA-silencing pathway can be considered to function in parallel for perception of VAMPs as a first layer of defense (Fig. 1).

### ETI‐based antiviral responses

In view of the above considerations, PTI was only recently included into virus–host interaction models, whereas ETI has long been recognized as an efficient plant defense mechanism against viruses. ETI is considered as a second layer of the plant innate immune system: effector proteins, delivered into host cells by adapted plant pathogens to suppress PTI, can become recognized by intracellular immune receptors in resistant host isolates. Effector recognition and immune receptor activation induce the rapid and efficient ETI response [6]. As an extreme output, ETI can culminate in the hypersensitive response, a programmed cell death reaction at infection sites thought to limit pathogen spread. Most intracellular immune receptors belong to the nucleotide binding/leucine-rich repeat (NLR) class of proteins reviewed in e.g. [21, 22]. Although the term effectors is not classically used in plant virus interactions, also viruses encode virulence factors or structural components, which can become recognized in an accession- and isolate-specific manner by the ETI system (reviewed in [23, 24]) (Fig. 1).

Over the past decade, several *R* genes that mediate dominant resistance against viruses have been identified (reviewed in [25]). Viral components recognized by R proteins are diverse, and include coat protein, movement protein, helicase or others. Plant NLRs are divided in two major groups based on their N-terminal coiled coil (CC) or Toll-like interleukin 1 receptor (TIR) domains, and are referred to as CNLs and TNLs, respectively [26]. Both these major classes of NLRs can function in resistance to viruses. Recent insights suggest that CNLs can induce ETI directly, by formation of a resistosome complex upon activation, which inserts into membranes to function as a Ca^2+^-permeable ion channel [27, 28]. By contrast, TNLs assemble into holoenzymes with NADase activity upon activation [29, 30]. Downstream of NADase activity, TNL immunity depends on ENHANCED DISEASE SUSCEPTIBILITY1 (EDS1) complexes and helper NLRs termed RNLs [31–34]. RNLs incorporate a subtype of the CC domain, the CC_R_ or CC_HeLo_ domain, with similarity to that of the non-LRR protein RESISTANCE TO POWDERY MILDEW 8 [26, 35]. While the CC_R_-type helper NLRs are relatively conserved, *TNLs* and *CNLs* belong to the most variable and most rapidly evolving gene families in plants [36–38]. ETI is induced only upon presence of an immune receptor in the host (encoded by the *R* gene) and the corresponding, recognized component in the pathogen (encoded by the *Avr* gene). Thus, ETI-mediated virus resistance generally is dominant and monogenic. Intracellular immune receptors can either recognize non-self as direct binders, or modified self as guardian NLRs [39, 40]. In the case of resistance to viruses, the details of pathogen recognition have been analysed in only few cases, as e.g. the recognition of the tobacco mosaic virus p50 helicase domain by the tobacco N receptor [41]. ETI against plant viruses often results in the hypersensitive response and extreme resistance, and is accompanied by phytohormone release [42]. By subsequent long-distance signalling, ETI therefore not only comprises the local response at the infection site, but also a systemic response conferring resistance to subsequent infections, known as systemic acquired resistance (SAR). SAR is conserved across different plant families and is non-specific. Therefore, it is effective against different pathogens (viruses, fungi, and bacteria) and may confer protection to subsequent infections.

## Main text

In this section we describe the main examples of host RNA-mediated regulation of virus resistance factors playing a role in PTI and ETI.

### RNA-based regulation of dicers: binding of VAMPs in analogy to PRRs

MicroRNAs (miRNAs) associate with Argonaute (AGO) proteins to direct widespread post-transcriptional gene repression. *Arabidopsis* miR393 was the first miRNA implicated in bacterial PTI [43]. The *miR393* gene is transcriptionally activated by flg22. High miR393 levels repress accumulation of the transcripts of the auxin receptor TRANSPORT INHIBITOR RESPONSE 1 (TIR1) and related AUXIN-SIGNALING F-BOX (AFB) proteins. This leads to reduced ubiquitination-mediated turnover of the auxin/indole-3-acetic acid (Aux/IAA) co-repressors, and thus suppression of auxin-responsive gene expression and PTI defenses. Indeed, bacterial growth was reduced in miR393 overexpressing *Arabidopsis* lines [43].

Similarly, TIR1-mediated Aux/IAA turnover and auxin signalling were found to be significantly affected in rice during infection by rice black-streaked dwarf virus (RBSDV) [44]. However, in contrast to flg22 recognition, miR393 is not upregulated in RBSDV-infected rice. This suggests that repression of auxin signalling by RBSDV may occur by a distinct mechanism not involving PRR-PAMP recognition. In the case of rice dwarf virus (an RBSDV relative), auxin signalling seems to be altered by the capsid protein P2 via a protein–protein interaction with at least one AUX/IAA, rather than by RNA-based mechanisms [45]. Accordingly, antiviral PRRs sensu stricto remain to be identified (although NIK1 may represent a valid candidate in this respect), and PRR involvement in antiviral PTI has been suggested only by indirect evidences.

**Fig. 2:**
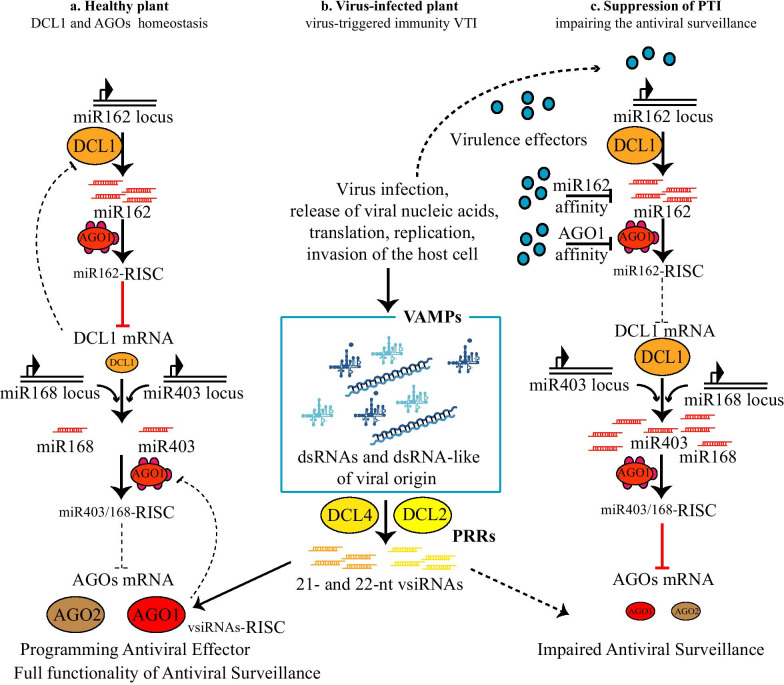
Schematic representation of miRNA-mediated regulation of antiviral DCLs and the action of virulence effectors. **a** In healthy plants, miR168 and miR403 regulate, at post-transcriptional level, the expression of Argonaute 1 and 2 (AGO1 and 2), respectively. miR168 and miR403 are processed by Dicer-like 1 (DCL1), which is, in turn, regulated at post-transcriptional levels by miR162. **b** Upon infection, VAMPs are perceived, and processed by the main antiviral DCLs, i.e., DCL2 and DCL4, into 22- and 21-nucleotide (nt) long virus-derived small-interfering RNAs (vsiRNAs). vsiRNAs can program AGO1 and 2, which thus assemble RISCs mediating full antiviral surveillance. **c** During viral infection, virulence effectors can alter homeostasis of DCL1, and in turn, of AGOs, mainly by sequestering miRNAs. This results in a suppression of PTI and AGO-mediated antiviral surveillance. Relative size of the elliptic symbols representing AGOs or DCLs are indicative of up/down regulation. Arrows indicate positive regulation and blunt-ended bars indicate inhibition. Black blunt-ended end bars indicate a virus effector affinity for siRNAs or for RISC. Dashed blunt-ended bars indicate mild inhibition compared to solid red lines

However, given that RNAi-mediated antiviral immunity acts as the major virus resistance mechanism in plants and exhibits PTI features [11], the main role of PRRs is played by plant DCLs (Figs. 1 and 2). Plant DCLs have specialized functions in producing short RNAs of 21- to 24-nucleotides (nt), including miRNAs and small-interfering (si)RNAs of endogenous or viral origin (vsiRNAs) [46]. DCL1-derived miRNAs modulate the expression of antiviral DCLs upon perception of viral infections (Fig. 2). As a consequence, antiviral DCL-derived vsiRNAs program antiviral effectors and confer antiviral immunity. In *Arabidopsis*, antiviral DCLs, *i.e*. DCL4 and DCL2, act redundantly in antiviral immunity: DCL4 and DCL2 are both sufficient for blocking the virus from spreading in plant tissues. However, viral suppressors of RNA-silencing (VSRs) reveal non-redundant functions and sub-specialization among DCL2/4 by specific inhibition of DCL4 activity [47, 48]. In vivo data revealed that miRNA sequestration by VSRs is most relevant at the early stage of an infection prior to the emergence of virus-induced symptoms or spread [49]. In the case of p19-deficient cymbidium ringspot virus, miR162 downregulates the expression of DCL1, which keeps the miRNA expression levels low and the DCL4 levels high. Conversely, early in infection, p19 binds miR403 and the expression of the antiviral AGO2 is increased, whereas the low affinity of p19 for *AGO1*-regulating miR168 ensures homeostasis of AGO1 [49] (Fig. 2).

**Fig. 3:**
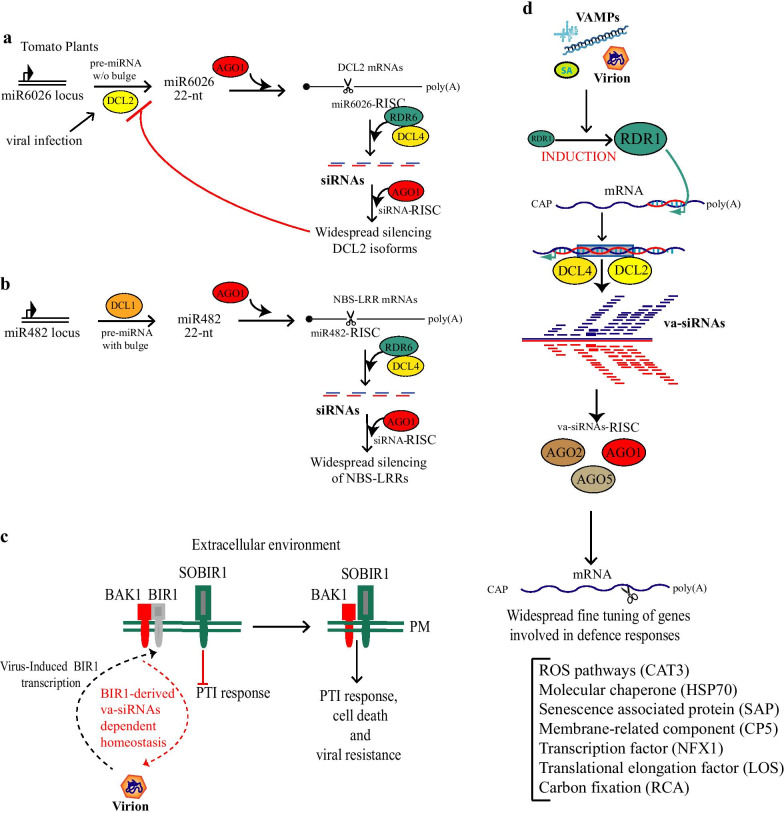
Regulatory RNA-silencing cascade and activation of endogenous siRNAs during viral infection. **a** Schematic representation of the non-canonical Dicer-like 2 (DCL2)-derived miR6026 function in the cleavage of DCL2 mRNA and in triggering secondary siRNAs and silencing cascade processes. **b** Schematic representation of the canonical Dicer-like 1 (DCL1)-derived miR482 function in the cleavage of mRNAs, which code for nucleotide binding/leucine-rich repeat-type immune receptors, and in triggering secondary siRNAs and silencing cascade processes. **c** Schematic representation of the transmembrane receptor-associated kinases SOBIR1 and BAK1, and the immune repressor BIR1. BIR1 contributes to antiviral defense and undergoes post-transcriptional regulation mediated by virus-activated (va) BIR1-derived secondary RNAs upon viral infection. **d** Schematic representation of vsiRNA biogenesis and widespread fine tuning of genes involved in defense responses. Upon infection, plant-encoded RNA-depended RNA polymerase 1 (RDR1) is induced, and drives the production of double-stranded (ds) RNAs using transcript as templates. Plant endogenous dsRNAs are processed by the induced antiviral DCL2 and 4 into a pool of vsiRNAs. vsiRNAs program AGO1, 2 and 5 to cleave mRNAs from where they are originated, and trigger silencing cascade mechanisms. Arrows indicate positive regulation and blunt-ended bars indicate inhibition. Blunt-ended end bars indicate an inhibitory effect. Plasma membrane (PM)

The basis for the DCL1-dependent negative regulation of DCL4 levels remains to be established. However, a novel regulatory mechanism of the antiviral DCL2 was recently revealed (Fig. 3a). DCL2 plays a key role in producing 22-nt sRNAs from endogenous mRNAs, viral RNAs and from transgenes when other DCLs, especially DCL4, are absent [46, 50–52]. DCL2 has other roles in the systemic spread of transitive silencing between cells and the vascular system [53, 54]. In tomato, DCL2 is the major dicer of defense against tobacco mosaic virus (TMV) and potato virus X, which is mediated by an unusual miRNA-dependent mechanism [55]. It is widely accepted that DCL1 typically produces 21-nt miRNAs, but generates 22-nt products if the precursor RNA has an asymmetric bulge in the base-paired region [56]. However, an alternative mechanism for production of 22-nt sRNA production involves DCL2, and is independent of bulges in the precursor RNA. E.g., in tomato, the biogenesis of non-canonical 22-nt miR6026 is DCL2-dependent [55]. Indeed, the miR6026 level is lower than for wild-type in *dcl2*-deficient plants, whereas the 22-nt miR482 level is unaffected except in *dcl1*-deficient lines [55] (Fig. 3). The same authors also showed that the *DCL2* transcript isoforms are among the miR6026 target mRNAs*.* In *Arabidopsis*, the 22-nt miRNAs trigger secondary sRNA production using their mRNA targets as template [57]. Similarly, in tomato, sRNAs corresponded to the miR6026 targets, were partly DCL4 and RDR6-dependent [55]. The antiviral activity of DCL2 was confirmed by the sRNA profiles of TMV-infected tomato: in *dcl2*-deficient plants, the 22-nt viral sRNAs were less abundant than in wild-type.

### RNA-based regulation of NLR genes

The RNA-silencing pathway further controls plant immune capacities via the ETI system. E.g., in tomato, levels of the NLR-type immune receptor slTM2, involved in antiviral ETI, are controlled by the 22-nt miR6026. Conversely to *DCL2* mRNAs, *slTM2* mRNA regulation is apparently not dependent on miR6026 targeting [55]. The authors attributed this to the fact that other miRNAs target the *slTM2* mRNA and could trigger secondary siRNA in the absence of miR6026.

Expression of *NLR* genes needs to be tightly controlled, as NLRs can also trigger autoimmunity in the absence of pathogen infection and inhibit plant growth; referred to as the “growth-defense trade-off” [58–60]. Therefore, plants have evolved sophisticated RNA-silencing-based cascade mechanisms to downregulate entire families of *NLR* genes, thus preventing autoimmunity, and opening the "gates" to establish symbiosis [61]. miRNAs are widely accepted as master regulators of mRNAs of the *NLR* immune receptor gene family via the production of phased secondary siRNAs [62–66]. In tomato, the initiators of the cascade are 22-nt-long miRNAs of the miR482 superfamily [67] (Fig. 3b). Members of this superfamily vary in sequence and abundance in different plant species, but generally target the coding sequence of the P-loop motif within the mRNA sequences of NLR immune receptors [67]. Oftentimes, due to natural variation within NLRs, miRNA-mediated cleavage is affected by indels in *NLR* alleles [65]. The targeting of *NLR* transcripts causes mRNA decay and RDR6-dependent production of phased secondary siRNAs in register with the cleavage site, which can regulate further transcripts upon incorporation into the RNA-induced silencing complex (RISC) [68]. At least one *NLR*-derived secondary siRNA was shown to act in *trans* and target mRNAs of additional defense-related proteins. Further, secondary siRNAs acting in *cis* fuel the cascade mechanism and boost the down-regulation of *NLR* genes. Viruses or bacteria can suppress sRNA-mediated *NLR* regulation by preventing miRNA incorporation into RISCs. As a result, a pathogen-induced expression of NLRs occurs and broad-spectrum defense mechanisms are activated [67, 69].

The requirement for dynamic regulation of *NLR* gene expression is further emphasized by characterization of miR1885 in *Brassica rapa* [70]. Here, miR1885 is not conserved, but appears to have recently evolved from an inverted duplication of an *NLR* gene. miR1885 expression is specifically induced upon TuMV infection, and regulates expression of highly sequence-related *TNL* genes [70]. However, miR1885 also targets the trans-acting silencing (TAS) gene *BraTIR1* [71]. In the absence of TuMV infection, miR1885 levels remain low to maintain normal development and basal immunity. Upon TuMV infection, repression of *BraTIR1* is entailed by repression of the photosynthesis-related gene *BraCP24*, which subsequently accelerates floral transition [71]. This sophisticated mechanism illustrates how plants, besides actively mounting defenses via the PTI, ETI and RNA-silencing pathways, invented alternative solutions to rapidly escape from virus infection by transitioning to a safe and healthy next generation.

### Widespread silencing of host genes and broad spectrum immunity

BAK1-INTERACTING RECEPTOR-LIKE KINASE 1 (BIR1) was identified as a negative regulator of plant immunity and cell death in a systematic reverse genetic screen, and shown to interact with the PRR co-receptor BAK1 [72]. However, BIR1 overexpression also leads to severe developmental defects and triggers the activation of plant defenses [73]. During tobacco rattle virus (TRV) infection in *Arabidopsis*, *BIR1* gene expression is activated in a partly salicylic acid-dependent manner, and is an important regulator of antiviral defenses [74]. Interestingly, siRNAs originating from *BIR1* mRNA were found to be produced during TRV infection and are involved in *BIR1* homeostasis [74] (Fig. 3c). Similar regulatory mechanisms may apply for additional plant immune components. Indeed, viral infections are accompanied by a massive production of siRNAs of plant origin, such as va-siRNAs, which drive the widespread silencing of host-gene expression [19, 20]. Data on *BIR1* regulation in the TRV-*Arabidopsis* system extend observations from cucumber mosaic virus (CMV) and TuMV-infected *Arabidopsis* and CaMV-infected *Brassicaeae*.

An important finding derived from the discovery of va-siRNAs is that the investigated *Brassicaceae* species display a highly conserved va-siRNA induction response during infection with taxonomically unrelated viruses [19, 20]. Moreover, the two antiviral dicers, DCL2 and DCL4, are mainly involved in the generation of va-siRNAs. va-siRNAs commonly target genes involved in biotic and abiotic stress response (Fig. 3d). Taken together, these observations support the notion that induction of antiviral silencing confers broad-spectrum antiviral activity as a result of widespread silencing of host genes (in particular negative regulators of defence responses), mediated by va-siRNAs in addition to specific antiviral defenses by vsiRNAs.

In the case of canonical PTI and PAMP perception, PRRs initiate signalling pathways leading to ROS production. Recent investigations (highlighted in [75]) have revealed that RNA-based mechanisms tune ROS pathways during plant virus infections. Interestingly, among the genes targeted by va-siRNAs and significantly down-regulated in CaMV-infected *Brassicaceae* and, similarly, in *A. thaliana* infected by CMV-∆2b (a CMV which lacks the 2b VSR), there were reactive oxygen species (ROS) scavenging factors such as Catalase 3 (Cat3), which could represent an element of parallelism with canonical PTI [20]. ROS pathways can also be regulated by viral proteins (i.e. VSRs), which then mask RNA-based regulatory mechanisms. The case of CAT3 is emblematic: the interaction between CMV 2b and CAT3 seemed to be important to induce necrosis, as a consequence of CAT3 degradation via the proteasome pathway. Furthermore, CMV accumulates more abundantly in plants that do not express CAT3 [76]. It therefore appears that viruses hijack the host's ROS generation mechanism during infection to promote viral replication by va-siRNAs-mediated gene regulation or viral protein-mediated CAT3 degradation. Another case worthy of note is that of a red clover necrotic mosaic virus protein that associates with positive regulators of the ROS production machinery. As a result ROS are induced by the plant and viral replication is more abundant [77]. In the same study, the authors have found a similar evidence for brome mosaic virus replication.

In rice infected with RSV, miR528 is suppressed at both the transcriptional and post-transcriptional levels. Furthermore, RSV induces the accumulation of the non-catalytic AGO18. AGO18 sequesters miR528 away from AGO1 to block the formation of an effective RISC. These events induce the accumulation of the miR528 target, L-ascorbate oxidase, thereby regulating cellular redox status and priming ROS-mediated resistance against RSV infection [78]. Based on the notion that ROS could limit virus invasion, the overexpression of negative regulators of ROS production could result in enhanced viral replication. *Triticum aestivum thioredoxin-like* gene (TaAAED1) encodes a negative regulator of ROS production in the chloroplast. Wheat yellow mosaic virus-derived dsRNAs are perceived and processed by DCL4 to produce 21-nt vsiRNAs. Upon incorporation into the RISC, the vsiRNAs suppress the expression of *TaAAED1* in a dose-dependent (more viral RNA, more downregulation) and sequence-specific manner. Therefore, chloroplast-generated ROS are known to induce retrograde signalling (from the chloroplast to the nucleus), leading to the modulation of the expression of pathogenesis-related genes that are involved in the defense response to viruses [79].

All together, these studies demonstrate that plant viruses are causal agents of ROS induction in infected plants; however, the function of ROS in plant-virus interactions remains unclear, because despite the intuitive idea that ROS are plant defence tools, in many cases intracellular bursts of ROS have been associated with increased viral fitness.

## Conclusions

Recent discoveries emphasize consistent parallels and connections between canonical plant immunity mechanisms (PTI, ETI) and the RNA-silencing pathway in orchestrating resistance to viruses and microbes. Especially the PTI system has been previously well-studied for its role and molecular functions in plant–microbe interactions, which may help to decipher its role in virus resistance in future. A most relevant finding is that DCLs may be the main actors, able to perceive viral PAMPs (VAMPs), in analogy to PRRs, and trigger resistance. These findings are extendable to mammalian cells where RNA detection mechanisms are well-established [80]. Recent insights offer important perspectives for bolstering plant defense against bacterial and fungal pathogens via RNAi approaches or applications. Studies of virus-host interactions show how RNA-mediated gene regulation can confer broad-spectrum resistance efficiently even towards pathogens that are routinely controlled by pesticides.

## Data Availability

Not available.

## Abbreviations

PTI: Pattern-triggered immunity
Avr: Avirulence proteins
ETI: Effector-triggered immunity
HR: Hypersensitive response
PRR: Pattern-recognition receptor
RLK: Receptor-like kinases
MAMPs/PAMPs: Microbe/pathogen‐associated molecular patterns
LRR: Leucine-rich repeat
FLS2: FLAGELLIN-SENSING 2 (FLS2)
ROS: Reactive oxygen species
NO: Nitric oxide
SA: Salicylic acid
DAMPs: Damage-associated molecular patterns (DAMPs)
BAK1/SERK3: BRASSINOSTEROID INSENSITIVE1 (BRI1)‐ASSOCIATED KINASE 1
SERKs: SOMATIC EMBRYOGENESIS RECEPTOR KINASES
VAMPs: Viral PAMPs
TuMV: Turnip mosaic virus
dsRNAs: Double-stranded RNAs
DCLs: Dicer-like proteins
NIK1: NSP-INTERACTING KINASE 1
va-siRNAs: Viral-activated siRNAs
R-proteins: Resistance proteins
NLR: Nucleotide binding/leucine-rich repeat
CC: Coiled coil
TIR: Toll-interleukin 1 receptor
CNLs: CC-NLRs
TNLs: TIR-NLRs
NADase: Nicotinamide adenine dinucleotide glycohydrolase
SAR: Systemic acquired resistance
miRNA: MicroRNA
AGO: Argonaute
TIR1: TRANSPORT INHIBITOR RESPONSE 1
AFB: AUXIN-SIGNALING F-BOX
Aux/IAA: Auxin/indole-3-acetic acid
RBSDV: Rice black-streaked dwarf virus
nt: Nucleotides
siRNAs: Small-interfering RNA
vsiRNAs: Viral-derived siRNA
VSR: Viral suppressor of RNA-silencing
TMV: Tobacco mosaic virus
RISC: RNA-induced silencing complex
TuMV: Turnip mosaic virus
TAS: Trans-acting silencing
BIR1: BAK1-INTERACTING RECEPTOR-LIKE KINASE 1
TRV: Tobacco rattle virus
CaMV: Cauliflower mosaic virus
TaAAED1: *Triticum aestivum thioredoxin-like* Gene
